# Increased expression of HIF2α during iron deficiency–associated megakaryocytic differentiation

**DOI:** 10.1111/jth.12884

**Published:** 2015-04-23

**Authors:** K. Jimenez, V. Khare, R. Evstatiev, S. Kulnigg‐Dabsch, M. Jambrich, H. Strobl, C. Gasche

**Affiliations:** ^1^ Division of Gastroenterology and HepatologyDepartment of Medicine IIIMedical University of ViennaViennaAustria; ^2^ Christian Doppler Laboratory on Molecular Cancer ChemopreventionMedical University of ViennaViennaAustria; ^3^ Center of Pathophysiology, Infectiology, and ImmunologyInstitute of ImmunologyMedical University of ViennaViennaAustria; ^4^ Center of Molecular MedicineInstitute of Pathophysiology and ImmunologyMedical University of GrazGrazAustria

**Keywords:** endothelial PAS domain‐containing protein 1, iron deficiency anemia, megakaryocytes, thrombocytosis, thrombopoiesis

## Abstract

**Background:**

Iron deficiency is associated with reactive thrombocytosis; however, the mechanisms driving this phenomenon remain unclear. We previously demonstrated that this occurs alongside enhanced megakaryopoiesis in iron‐deficient rats, without alterations in the megakaryopoietic growth factors thrombopoietin, interleukin‐6, or interleukin‐11.

**Objectives:**

The aim of this study was to evaluate megakaryocyte differentiation under iron deficiency in an *in vitro* model and to investigate potential genes involved in this process.

**Methods:**

Human erythroleukemia and megakaryoblastic leukemia cell lines, as well as cord‐blood derived hematopoietic stem cells were cultured under iron deficiency. Cell morphology, ploidy, expression of CD41, CD61, and CD42b, and proplatelet formation were assessed in iron‐deficient cultures. Polymerase chain reaction arrays were used to identify candidate genes that were verified using real‐time polymerase chain reaction. Hypoxia‐inducible factor 1, α subunit (HIF2α) protein expression was assessed in bone marrow sections from iron‐deficient rats and vascular endothelial growth factor (VEGF)‐A in culture supernatants.

**Results and Conclusions:**

Iron deficiency enhanced megakaryoid features in cell lines, increasing ploidy and initiating formation of proplatelet‐like structures. In cord blood cell cultures, iron deficiency increased the percentage of cells expressing megakaryopoietic markers and enhanced proplatelet formation. HIF2α and VEGF were identified as potential pathways involved in this process. HIF2α protein expression was increased in megakaryocytes from iron‐deficient rats, and VEGF‐A concentration was higher in iron‐deficient culture supernatants. Addition of VEGF‐A to cell cultures increased percentage expression of megakaryocyte CD41. In conclusion, the data demonstrate that iron deficiency augments megakaryocytic differentiation and proplatelet formation and a potential role of HIF2α in megakaryopoiesis.

## Introduction

Platelets play a fundamental role in hemostasis and thrombosis and an emerging role in inflammation and cancer biology. Platelet production occurs at a rate of 10^11^ platelets daily and may increase up to 20‐fold in response to high demand 1. Reactive thrombocytosis occurs in response to infection, tissue damage as occurs during surgery, chronic inflammation, malignancy, and post splenectomy 2 and is by far more common than primary thrombocytosis. Another well‐known cause of reactive thrombocytosis is iron deficiency (ID) 3, 4, 5, 6. While the elevation of platelet count is usually mild to moderate in ID (~ 500 × 10^9^ L^−1^), platelet counts reaching 1000 × 10^9^ L^−1^ have been reported 3, 4.

The mechanism behind this phenomenon is not fully elucidated; however, animal models of ID―including a diet‐induced ID model established by our group―show that ID alone leads to increased platelet count 7, 8, 9. This is accompanied by changes in megakaryopoiesis, such as enhanced progenitor expansion, increased megakaryocyte (MEG) ploidy, and overall augmented MEG differentiation. Furthermore, platelets produced in ID had higher aggregability 9, suggesting that elevated platelet count in ID may not be entirely risk free. There are numerous case reports linking ID‐related thrombocytosis and thrombosis 10 and several case–control studies showing that ID is more common in patients with a cerebrovascular insult in comparison to controls 11, 12, 13.

Anemia is a common complication in inflammatory bowel disease (IBD) (6.5–73.7%) 14 and in cancer (30–90%) 15; in both cases, ID appears to be the predominant cause 14, 15, 16, 17. Thrombocytosis is not uncommon in either condition 18, 19, and both cancer and IBD patients have increased risk for venous thromboembolism (VTE) 20, 21, 22, 23, for which, in the case of cancer, high platelet count is an independent risk factor 20, 21. Iron replacement therapy in patients with IBD and ID anemia normalizes platelet count and reduces platelet activity 24, 25. While it is unclear if this affects risk for thromboembolic events in the long term, anemic cancer patients have a diminished incidence of VTE if erythropoiesis‐stimulating agents are administered with intravenous iron 26 as opposed to no‐iron therapy. This suggests that ID‐related thrombocytosis may be of relevance in the clinical setting as well.

Megakaryocytes arise from hematopoietic stem cells, and share a common progenitor with erythrocytes 27. Megakaryocyte differentiation is marked by augmented expression of CD41, CD61, and CD42b, while erythroid markers such as glycophorin A are downregulated 27. Megakaryocytes undergo endomitosis, initiating multiple cycles of DNA replication without completing cytokinesis 28, allowing ploidy to increase geometrically from 2n to 64n. This functional gene amplification facilitates the ensuing increase in protein and lipid synthesis and substantial cell enlargement required for platelet biogenesis and function 29. An MEG may produce platelets at any ploidy level, although higher ploidy level may correlate with higher platelet production 30. Megakaryocyte terminal differentiation involves migration from the hypoxic osteoblastic niche toward bone marrow sinusoids 31, where cytoplasmic projections termed proplatelets are extended into the bloodstream and then fragment into platelets (reviewed in 32).

Iron is critical for cell survival, growth, and differentiation and is a functional component of heme‐ and iron‐sulfur‐cluster proteins involved in mitochondrial function, catalysis, redox reactions, DNA replication, and transcription (reviewed in 33). There is not much known about iron homeostasis in MEGs and how ID affects megakaryopoiesis. Cytokines more commonly associated with mediating megakaryopoiesis such as thrombopoietin, interleukin (IL)‐6, and IL‐11 are not altered in patients with ID anemia and thrombocytosis 24, 34 or in our animal model of ID 9.

In this study, we investigated potential targets that may be involved in ID megakaryopoiesis in an *in vitro* model of ID. ID enhanced MEG features in cell lines and in cord blood–derived hematopoietic stem cells (CBHSCs) and modulated the genes involved in the hypoxia‐inducible factor (HIF) and vascular endothelial growth factor (VEGF) pathways.

## Methods

### Culture of cell lines

Human erythroleukemia (HEL) and megakaryoblastic leukemia (CMK) human cell lines (DSMZ, Braunschweig, Germany) were maintained in RPMI (Gibco/Life Technologies, Lofer, Austria) and adapted to serum‐free and iron‐replete (IR) medium (Panserin 401; PanBiotech, Aidenbach, Germany) before experiments. For concentration gradients, IR medium was mixed with serum‐, iron‐, and transferrin‐free media (Panserin 401S; PanBiotech) to produce 100%, 10%, 5%, 2.5%, 1.25%, and 0% v/v concentrations. Subsequent experiments were performed with 100% (IR) and 1.25% (ID). Medium was not changed during experiments, so as not to reintroduce iron into ID cultures. Megakaryocytic differentiation was induced using 50 nmol l^–1^ phorbol‐12‐myristate‐13‐acetate (PMA; Sigma‐Aldrich, Munich, Germany) in IR medium. Cells were kept at 37°C, with 5% CO_2_ and a fully humidified atmosphere.

Cell proliferation was assessed through flow cytometric measurement of propidium iodide (PI; Sigma‐Aldrich) excluding cells and ploidy via Hoechst 33342 (Invitrogen/Life Technologies, Lofer, Austria) on a Cell Lab Quanta SC flow cytometer (Beckman Coulter, Vienna, Austria). Histograms were divided into 2n, 4n, and greater than 4n (> 4n) ploidy. Morphological images (×40) were taken with use of an Olympus IX81 inverted microscope, and DAPI (Vectashield; Vector Laboratories, Peterborough, UK)‐stained nuclei (×40) were photographed by using an Olympus BX51 microscope.

### CBHSC isolation and culture

Cord blood was obtained from the Department of Obstetrics and Feto‐Maternal Medicine after approval by the Ethics Commission of the Medical University of Vienna. CD34^+^ CBHSCs were isolated by using MACS (Miltenyi Biotec, Bergisch Gladbach, Germany), cultured for 6–7 days in serum‐free medium (X‐vivo 15; Lonza, Szabo, Vienna, Austria) with 50 ng mL^−1^ thrombopoietin (TPO; Kyowa Hakko Kirin Co., Ltd., Tokyo, Japan), and transferred to IR (100%) or ID (1.25%) serum‐free medium with 100 ng mL^−1^ TPO for 5 days.

Megakaryocyte differentiation was assessed by flow cytometric measurement of CD41, CD61, and CD42b surface expression (see Data S1). The percentage of positive staining cells was determined using flow cytometry, and the median fluorescence intensity (MFI) was based on the gated positive cell population of each MEG marker. Ploidy was measured as in cell lines, with anti‐CD41 to gate for MEGs. VEGF‐A in cell supernatants was assessed via immunoassay (ProcartaPlex, eBioscience, Vienna, Austria) measured on a BioPlex 200 (BioRad; Hercules, CA, USA). Cell counts after culture in IR and ID media supplemented with 25 ng mL^−1^ VEGF (eBioscience), 1 ng mL^−1^ erythropoietin (EPO; eBioscience), or 100 ng mL^−1^ TPO were performed after staining with anti–CD41‐FITC (MEG) and anti–glycophorin A‐PE‐Cy5 (erythrocyte) conjugated antibodies (eBioscience). At least 100 cells per condition were counted under ×40 magnification. To calculate percentages, the number of green (CD41) or red (glycophorin A) cells were divided by the total number of visible nuclei (see Data S1).

### Proplatelet formation

Proplatelet formation and assessment at day 5 were based on previously described methods 35, 36 (see Data S1). Fixed samples were incubated with mouse anti–α‐tubulin (Sigma‐Aldrich) followed by anti–mouse‐IgG conjugated to Alexa488 (Invitrogen/Life Technologies), and DAPI for nuclear counterstaining. Images (×10) were taken with an Axioimager M2 and analyzed using Zen lite software (Zeiss, Munich, Germany; Fig. S1).

### Real‐time polymerase chain reaction analysis of gene expression

Real‐time polymerase chain reaction (RT‐PCR) was performed using a TaqMan array with customized targets and a catalogued array for transcription factors (Applied Biosystems/Life Technologies, Lofer, Austria). RNA was pooled from two independent experiments after 1 day (HEL) or 2 days (CMK) of culture in ID (slower growth of CMK compared with HEL). Three independent 5‐day experiments of CD61^+^ MACS sorted MEGs were pooled. HPRT1 and 18S were used as endogenous controls, and cut‐offs were set at 2.0 for upregulation and 0.5 for downregulation. Individual genes such as *TfR1, HIF2α*,* HIF1α*,* VEGFA* (Qiagen, Hilden, Germany), *VEGFR1*, and *VEGFR2* (VBC Biotech, Vienna, Austria) were verified using SYBR green (Applied Biosystems/Life Technologies). Analysis was performed using Applied Biosystems software and LinRegPCR 37. Relative expression was calculated using $2^{-(CT_{\mathrm{Targe}t^{-},}CT_{\mathrm{Housekeeping}})}$ and relative quantity using $2^{-(\Delta CT_{\mathrm{ID}}-\Delta CT_{\mathrm{IR}})}$.

### Immunohistochemical staining

DAB‐HRP staining was performed on paraffin‐embedded sections of rat sterna from the rat model of ID 9 after incubation with anti‐HIF2α (Novus Biologicals, Abingdon, UK). Images of slides with covered labels were randomized and evaluated in ImageJ. Individual MEGs were evaluated based on staining intensity from 0 (no staining) to 3+ (intense staining). Immunohistochemical score was calculated by summing the product of percentage total MEGs and score number. Blood was drawn from the sublingual vein and analyzed on a Cell‐Dyn 3500 analyzer (Abbott Diagnostics, Abbot Park, IL, USA).

### Statistical testing

Statistical testing was performed using *t*‐test for independent samples, one‐sample *t*‐test with a test value of 1 for fold changes, anova with Tukey post‐hoc testing, and the Mann–Whitney *U* Test in SPSS 21.

## Results

### Iron deficiency induces megakaryocytic differentiation in HEL and CMK

HEL and CMK cell lines were selected because both are capable of megakaryocytic differentiation on induction by PMA 38, 39, 40. HEL is derived from a patient with erythroleukemia 41 and is less mature in comparison to CMK, which is derived from a patient with megakaryoblastic leukemia 42, thus allowing insight into different stages of megakaryopoiesis.

**Figure 1:**
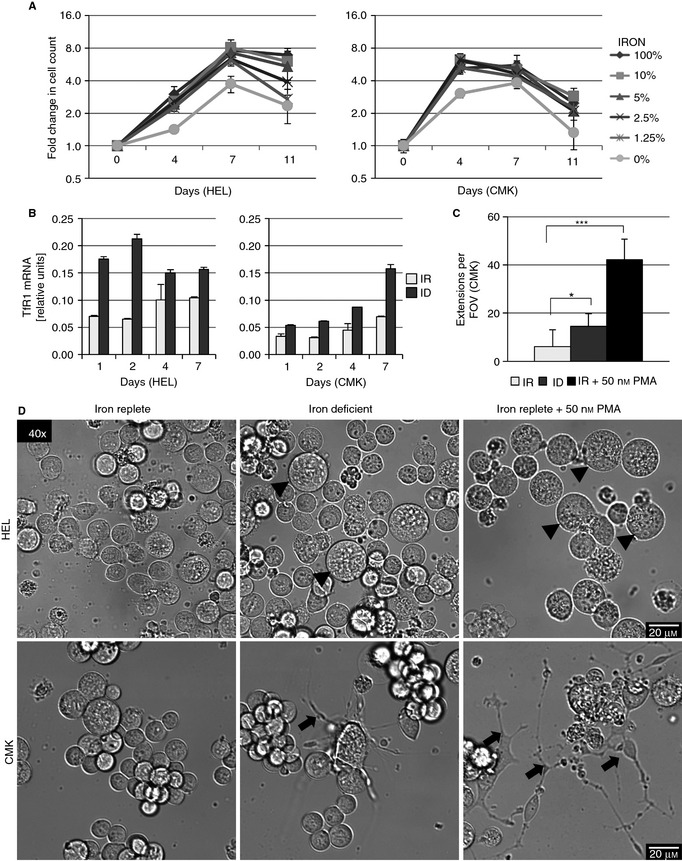
Iron deficiency (ID) leads to megakaryocytic morphological changes in HEL and in CMK. (A) Proliferation of HEL and CMK cells on culture in 100% (iron replete [IR]), 10%, 5%, 2.5%, 1.25% (ID), and 0% v/v Panserin 401 in Panserin 401S after 4, 7, and 11 days of culture. Graphs depict the fold change in cell count of propidium iodide–excluding cells measured by flow cytometry at each condition and time point as compared with day 0. (B) Relative TfR1 mRNA concentration of HEL and CMK cultured in IR or ID medium for 1, 2, 4, and 7 days (normalized to GAPDH). TfR1 is stabilized under low iron conditions. (C) Mean count of proplatelet extensions per ×40 field of view in CMK after culture for 4 days in IR, ID, and IR medium with 50 nmol L^–1^ PMA. (D) Representative pictures of live cell imaging (×40) of HEL and CMK cultured in IR, ID, and IR medium plus 50 nmol L^–1^ PMA for 4 days. Arrowheads depict large cells, which increase in ID and PMA‐treated HEL, while arrows depict formation of proplatelet‐like structures in CMK. The results from two or three independent experiments are shown. *P ≤ 0.05, ***P ≤ 0.001.

Culture in media without iron led to diminished proliferation in both HEL and CMK in comparison to fully IR media (Fig. 1A), and area under the curve (AUC) analysis confirmed this difference (Fig. S2A). Proliferation was arrested at day 11 and thus excluded from analysis. The 1.25% condition was selected for ID as it allows for cell proliferation, while still rendering cultures iron deficient. *TfR1*, which is stabilized when iron is low 43, is upregulated in ID in both cell lines on RT‐PCR (Fig. 1B), beginning after 1 day of culture and reaching a maximum after 2 days in HEL and 7 in CMK. Flow cytometric measurement showed an increased surface *TfR1* expression in HEL and CMK; however, the differences were not as distinct as on RT‐PCR (Fig. S2B).

**Figure 2:**
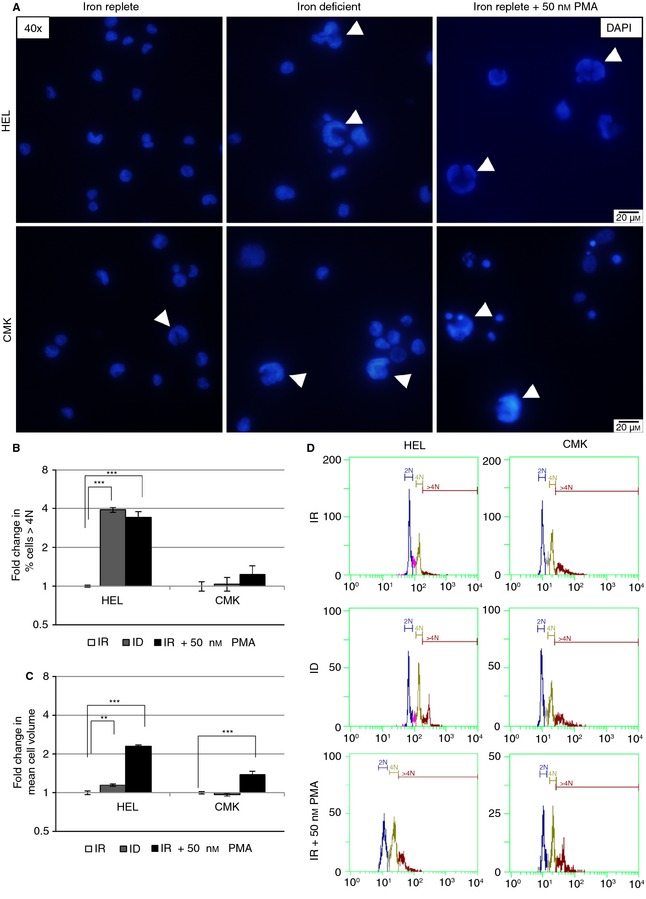
Iron deficiency (ID) increases ploidy in HEL. HEL and CMK cultured for 4 days in iron‐replete conditions (IR), ID, and IR plus 50 nmol L^–1^ PMA. (A) Representative images (×40) of nuclear staining (DAPI) of HEL and CMK after culture for 4 days in IR, ID, and IR media with 50 nmol L^–1^ PMA. Arrowheads depict large nuclei. (B) Flow cytometric measurement of Hoechst 33342 nuclear staining of HEL and CMK. Graphs depict the percentage of cells with ploidy greater than 4n as a fold change to IR. (C) Flow cytometric assessment of mean HEL and CMK cell volume depicted as fold change to IR. (D) Representative histograms of ploidy in HEL and CMK after 4 days’ culture in IR, ID, and IR + 50 nmol L^–1^ PMA. The results from two or three independent experiments are shown. **P ≤ 0.01, ***P ≤ 0.001.

Culture in ID resulted in distinct morphological changes similar to those observed on megakaryocytic differentiation by PMA (Fig. 1D). Cell volume was increased in HEL after 4 days of culture in ID (Fig. 1D, arrowheads; Fig. 2C, *P* = 0.008) and IR plus 50 nmol L^–1^ PMA (*P* < 0.001) compared with IR alone (Fig. 1D, arrowheads). In CMK, volume was increased in PMA treatment (*P* = 0.001) but not in ID (*P* = 0.95).

The percentage of HEL cells with a ploidy greater than 4n increased almost 4‐fold on flow cytometry (Fig. 2B, D). This matches the larger nuclei seen in fluorescence microscopy (Fig. 2A). Similar changes occurred in HEL treated with 50 nmol L^–1^ PMA. CMK, in contrast, did not increase in ploidy under ID and showed only a trend to increased ploidy on PMA treatment and no apparent differences in nuclear size. These changes in ploidy in ID corroborate previous data 9. In addition, proplatelet‐like cytoplasmic extensions formed in CMK under ID (*P* = 0.01, Fig. 1C, D, arrows), which were similar to the changes observed on treatment with 50 nmol L^–1^ PMA (*P* < 0.001). Addition of 50 nmol L^–1^ PMA to ID media did not show additive effects in ploidy or cell volume in HEL (Fig. S3C) and was lethal in CMK, preventing analysis (Fig. S3A). Thus, ID may influence different aspects of megakaryopoiesis, augmenting endomitosis and later events such as proplatelet formation.

### Iron deficiency potentiates MEG differentiation in CBHSCs

**Figure 3:**
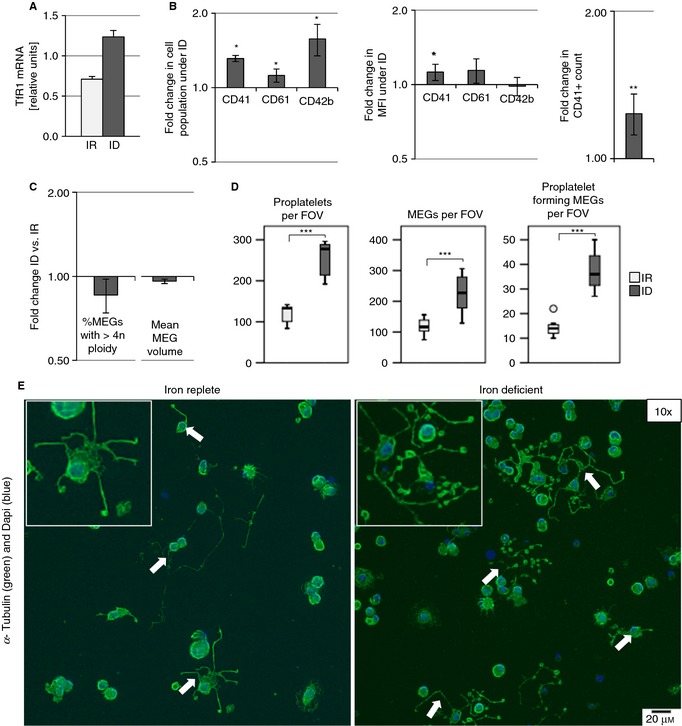
Iron deficiency (ID) potentiates megakaryocyte (MEG) differentiation in cord blood–derived hematopoietic stem cells. MEGs cultured in iron‐replete (IR) and ID media supplemented with 100 ng mL^−1^ TPO for 5 days. (A) Relative TfR1 mRNA concentration of MEGs cultured in IR and ID media (normalized to HPRT1). (B) Flow cytometric measurement of CD41, CD61, and CD42b after MEG culture in ID and IR media. Graphs depict the fold change in the percentage of cells expressing surface markers; the fold change in the median fluorescence intensity (MFI) of the gated positive CD41, CD61, or CD42b cells; and the fold change in the CD41^+^ cell count in ID‐ vs. IR‐cultured MEGs. (C) Flow cytometric measurement of ploidy by Hoechst 33342 nuclear staining. Graph depicts the fold change in the percentage of MEGs with ploidy greater than 4n and the mean MEG volume in ID compared with IR. (D) Boxplots depict proplatelet count per field of view (FOV), MEG number per FOV, and number of MEGs forming proplatelets per FOV after culture in ID and IR media. (E) Representative images (×10) of adherent MEGs forming proplatelets on fibrinogen‐coated coverslips after 5 days’ culture in IR and ID media. Arrows indicate proplatelet extensions. Insets show zoomed‐in proplatelet structures. The results from three to five independent experiments are shown. *P ≤ 0.05, **P ≤ 0.01, ***P ≤ 0.001.

To investigate megakaryopoiesis under ID in primary cells, CBHSCs were cultured in IR or ID medium. Induction of ID was confirmed by increased *TfR1* expression on RT‐PCR for *TfR1* (Fig. 3A).

The percentage of CD41‐expressing cells increased 1.31 ± 0.04‐fold (*P* = 0.001) in ID compared with IR. Similarly, the percentage of CD61 (1.12 ± 0.07‐fold, *P* = 0.017) and CD42b (1.58 ± 0.23‐fold, *P* = 0.49) were increased in ID (Fig. 3B, S4A). Median fluorescence intensity for CD41 within the CD41‐positive population also increased. These results show a shift toward the MEG lineage, which progressively upregulates CD41 and later expresses CD61 and CD42b. Furthermore, the concentration of cells expressing CD41 increased by 1.24 ± 0.08‐fold (*P* = 0.009, Fig. 3B), unlike the cells that were CD41 negative (Fig. S4B). Unlike in cell lines, ploidy and cell volume did not increase (Fig. 3C, Fig. S4C, E).

ID increased the total number of proplatelets formed, the total number of cells that adhered to the fibrinogen‐coated slide, and the number of cells actively forming proplatelets (Fig. 3D). The ratio of proplatelet‐forming MEGs to total MEGs had a trend toward increase under ID (Fig. S4D). ID did not appear to increase the number of proplatelets formed per MEG (Fig. S4D).

### Iron deficiency differentially regulates gene expression in HEL, CMK, and MEGs

Custom array targets were selected to include genes involved in iron homeostasis as well as hematopoiesis, megakaryopoiesis, and erythropoiesis. Cell cycle regulators were also included, because ID may influence endomitosis. We included genes involved in apoptosis, because ID influences apoptosis and platelet production is influenced by apoptotic pathways. Inclusion of genes involved in the generation of reactive species and antioxidant enzymes was based on the role of iron in oxidative stress (reviewed in 44). We were interested in the HIF pathway regulating response to hypoxic stress, which occurs during ID anemia and is potentially regulated by iron. HIF2α, in particular, is involved in the regulation of erythropoiesis under hypoxia and regulates targets involved in iron homeostasis (reviewed in 45). We also used a second preassembled array for transcription factors (Table S1).

**Figure 4:**
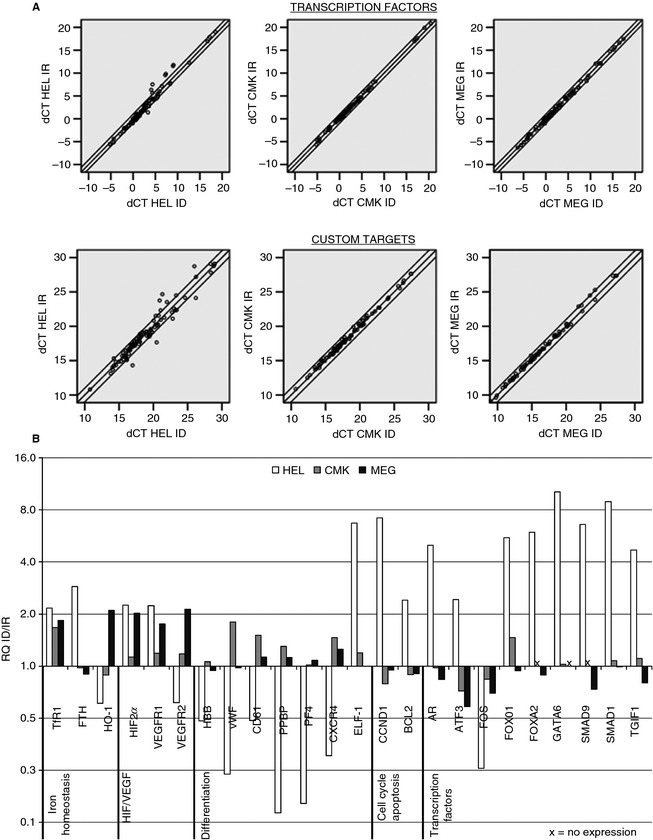
Iron deficiency (ID) alters gene expression. Real‐time quantitative PCR analysis of custom targets and transcription factors in HEL, CMK, and megakaryocytes (MEGs) after 1, 2, and 5 days of culture, respectively. (A) Iron‐replete (IR) Δ*C*_t_ plotted against the ID Δ*C*_t_ of HEL, CMK, and MEGs after analysis of transcription factors and custom targets gene expression. Double lines represent thresholds of 0.5 and 2.0. (B) Relative mRNA quantity of genes that reached the set cutoffs of 0.5‐ and 2.0‐fold changes in at least one cell type under ID. Genes that were not detected are marked with *x*. Data were obtained from samples pooled from two or three independent experiments. FOV indicates field of view.

Of 187 genes from the RT‐PCR arrays, only 23 were found to be regulated in HEL, none in CMK, and three in MEGs (Fig. 4, Table S1). *TfR1* was increased in all three cell types in ID; however, only HEL reached the set cut‐off, which indicates this cut‐off is stringent (Fig. 4B). Of the regulated targets, only *HIF2α* was commonly upregulated in HEL and MEGs above 2‐fold. *VEGF‐R1* was increased in both HEL and MEGs; however, the latter only had a 1.75‐fold change. *VEGF‐R2* was upregulated in MEGs but downregulated in HEL.

Apart from *TfR1*, several genes involved in iron homeostasis were regulated by ID. Heme oxygenase 1, which is inducible and previously identified as strongly responsive to ID and oxidative stress 46, was downregulated in HEL but upregulated in MEG (Fig. 4B). Heavy chain ferritin *(FTH*) and *SMAD1*, a regulator of hepcidin, a key player in iron homeostasis 33, were also upregulated in HEL.

In HEL, transcription factors involved in hematopoietic and megakaryocytic differentiation such as *TGIF1* 47, *ELF‐1* 48, 49, and *GATA‐6* 48, were upregulated, while *FOS* was downregulated. Treatment of HEL cells with testosterone increases the expression of thromboxane A2 receptor 50, suggesting that the ID‐induced increase in androgen receptor may have consequences for platelet function (Fig. 4B). Antiapoptotic gene *BCL2* may influence cell survival and transition from endomitosis to platelet production. The specific roles of *ATF3, FOXO1, FOXA2,* and *SMAD9* in the context of ID‐megakaryopoiesis are unclear.

In CMK, the expression of *CD61* (1.51‐fold change) and von Willebrand factor (1.80‐fold change) increased but did reach the 2.0 cut‐off. In HEL, cyclin D (*CCND1*) was highly upregulated, and genes involved both in erythroid (*HBB*) and megakaryoid differentiation (*VWF, CD61, PF4, PPBP*) were downregulated (Fig. 4B).

We were interested in the regulation of *HIF2α, VEGFR1*, and *VEGFR2* by ID because HIF2α coordinates oxygen and iron status with erythropoiesis and the VEGF pathway has been shown to influence various aspects of megakaryopoiesis 51, 52, 53. Of the identified targets, only these were similarly regulated in HEL and MEGs. Additionally, VEGF is under HIF regulation.

**Figure 5:**
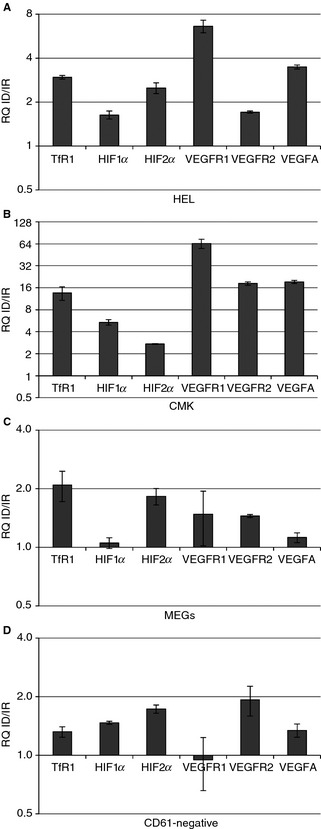
Regulation of genes in the hypoxia‐inducible factor (HIF) and vascular endothelial growth factor (VEGF) pathways is retained in subsequent experiments with iron deficiency (ID). Verification of selected identified regulated genes via real time quantitative PCR (RT‐PCR) in human erythroleukemia (HEL) (A) and megakaryoblastic leukemia (CMK) (B) after 3 days and megakaryocytes (MEGs) (C) and CD61‐negative cells (D) after 5 days of ID and iron‐replete condition (IR). Graphs depict the relative mRNA quantity (RQ) of selected genes in cells cultured under ID as compared to IR. All results are normalized to *HPRT1*.

To verify the expression of genes in HIF/VEGF pathway in ID, HEL and CMK were cultured under ID for 3 days and CBHSCs for 5 days prior to RT‐PCR (Fig. 5). *TfR1* was upregulated in ID in all cell types. *HIF2α* was also upregulated in all cell types. *VEGFR1* was upregulated in HEL, and CMK but not in MEGs and CD61‐negative cells. *VEGFR2* was upregulated in HEL, CMK, and MEGs but not in CD61‐negative cells. *VEGFA*, a known downstream target of the HIF pathway and ligand to these receptors 54, 55, was increased in all cell types, despite not being identified in the previous screen*. HIF1α* was upregulated in HEL, CMK, and CD61‐negative cells but not in MEGs. Overall, the data suggest a role for HIF2α and VEGFA in megakaryocytic differentiation in ID.

**Figure 6:**
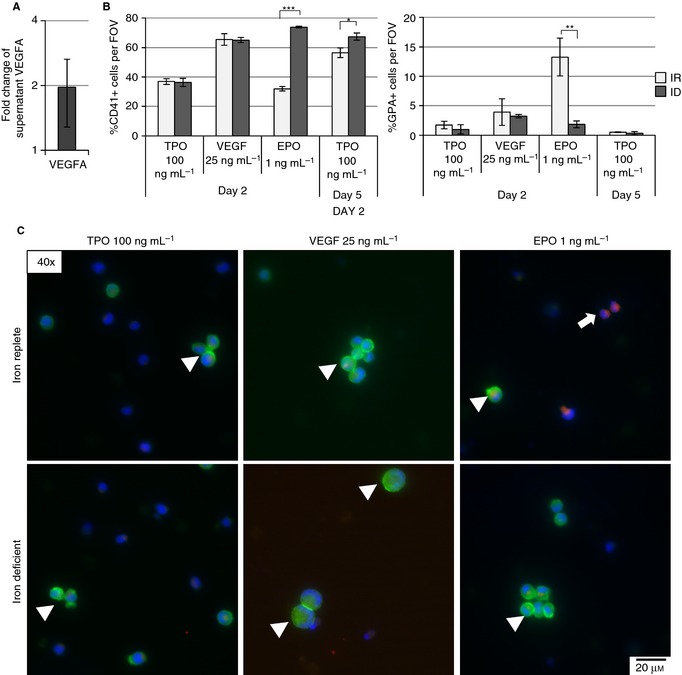
VEGFA increases megakaryocyte (MEG) concentration in cord blood–derived hematopoietic stem cells. (A) Measurement of VEGFA concentration in MEG culture supernatants after 5 days of iron deficiency (ID) by immunoassay. Graphs depict the fold change in the concentration of VEGFA of ID vs. iron‐replete (IR) media. (B) Mean percent of CD41^+^ and glycophorin A (GPA)^+^ cells per field of view (FOV) of stained and fixed samples after 2‐day culture in IR and ID media supplemented with 100 ng mL^−1^ TPO (control), 25 ng mL^−1^ VEGF, and 1 ng mL^−1^ EPO. (C) Representative images of CD41 (green, arrowheads), glycophorin A (red, arrows), and DAPI nuclear staining after culture in IR and ID media supplemented with 100 ng mL^−1^ TPO (control), 25 ng mL^−1^ VEGF, and 1 ng mL^−1^ EPO for 2 days. The results from two to five independent experiments are shown. *P ≤ 0.05, **P ≤ 0.01, ***P ≤ 0.001.

Because previous studies have shown that VEGF‐A influences megakaryopoiesis 51, 52, 53 and because VEGF‐A is a known target of HIF2α 54, 55, we evaluated the concentration of VEGF‐A in the cell media of MEGs. The concentration of VEGF‐A in media from ID cultures increased almost 2‐fold (Fig. 6A). When cells were cultured (48 hours) in IR and ID media supplemented with VEGF‐A (25 ng mL^−1^), the percentage of CD41+ cells per field of view increased in VEGF‐treated cultures in comparison to TPO‐treated cultures, but not the percentage of GPA+ cells (Fig. 6B, C). EPO (1 ng mL^−1^)‐supplemented cultures had a higher concentration of GPA+ cells in IR compared with ID (*P* = 0.004); conversely, CD41^+^ cell concentration was higher in ID (*P* < 0.001). While we did not see changes between TPO‐treated ID and IR cultures at this early timepoint, CD41^+^ cell concentration was increased after 5 days of culture (*P* = 0.029, Fig. 6B, Fig. S5). EPO and VEGF‐A cultures did not survive up to day 5, preventing analysis at this timepoint. These results support ID‐driven megakaryopoiesis, where EPO exacerbates ID leading to a higher percentage of CD41^+^ cells. Furthermore, the increase in percentage CD41^+^ with VEGF in comparison to TPO and the lack of difference between ID and IR suggest that VEGF may have a role in this process.

### HIF2α expression is increased in iron deficiency and correlates with platelet count

**Figure 7:**
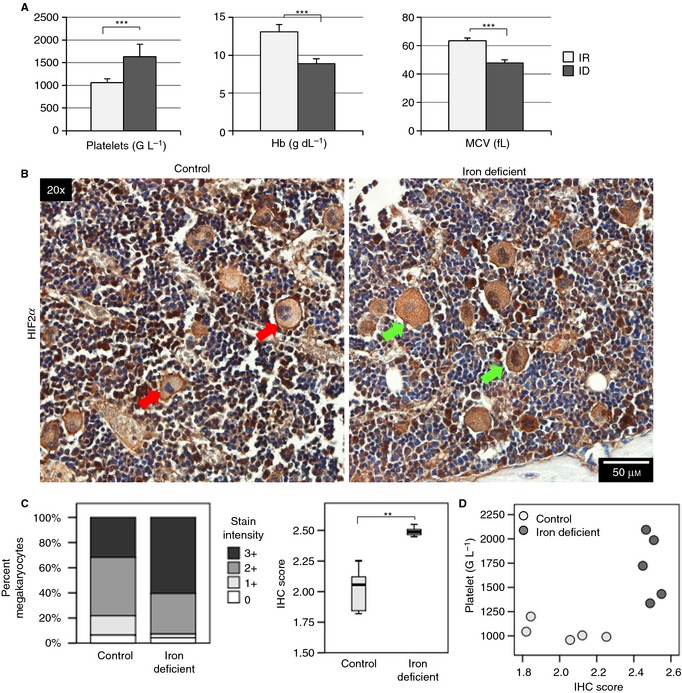
HIF2α expression is increased in iron‐deficient (ID) bone marrow megakaryocytes. (A) Platelet count, hemoglobin (Hb), and mean corpuscular volume (MCV) of control (*n* = 7) and iron‐deficient (*n* = 8) rats after 3 weeks. Analysis was performed on a Cell‐Dyn 3500 analyzer. (B) Representative images of bone marrow IHC for HIF2α from control and iron‐deficient rats. Red arrows depict megakaryocytes in control mice with less‐intense HIF2α staining, and green arrows depict megakaryocytes in ID mice with more‐intense staining. (C) Percentage of total control (*n* = 1735) and iron‐deficient (*n* = 1621) bone marrow megakaryocytes under each staining intensity and the median calculated IHC score for control (*n* = 5) and iron‐deficient (*n* = 5) rats. (D) Scatterplots of platelet count against IHC score. ***P ≤ 0.001. FOV indicates field of view.

To determine if protein levels of HIF2α were increased *in vivo* as well, we used sternal sections of ID and control rats from our previous experiments 9 for IHC analysis. In this animal model, maintenance on a diet low in iron over the course of 3 weeks resulted in increased platelet counts (*P* < 0.001), as well as diminished hemoglobin (Hb, *P* < 0.001) and mean corpuscular volume (MCV, *P* < 0.001) (Fig. 7A), which corresponds with the microcytic hypochromic anemia induced by ID in humans. Erythrocyte count and hematocrit were likewise diminished in ID (Fig. S6A), and leukocyte count was unchanged (Fig. S6A). HIF2α was present in MEGs (Fig. 7B, red arrows) and non‐MEGs in control rats, while staining intensity was diminished in non‐MEGs in most sections and increased in MEGs in ID rats (Fig. 7B, green arrows, see Fig. S6B for isotype control). IHC scoring confirmed increase in staining intensity upon ID (*P* = 0.008) as compared with control rats (Fig. 7B, C). Plots of platelet counts against IHC score show two separate clusters corresponding to control and ID animals (Fig. 7C) and indicate increased HIF2α with higher platelet counts in ID.

## Discussion

In this study, we utilized various *in vitro* models to examine the effect of ID on megakaryopoiesis and to identify potential targets involved in this process. We show that ID induces megakaryocytic differentiation and proplatelet formation. The novel finding of this study is identification of the HIF/VEGF pathways as potential regulators of megakaryocytic differentiation under ID. Apart from *TfR1*, the expression of genes from the HIF and VEGF pathways were identified by RT‐PCR arrays to be induced upon ID. The induction of HIF2α was verified in bone marrow of ID animals as was the secretion of VEGF‐A in supernatants of CBHSC. VEGF‐A supplementation increased MEG percentages regardless of iron concentration, suggesting involvement in iron deficient megakaryopoiesis.

Our lab has previously demonstrated increased ploidy in iron deficient megakaryocytic cell lines 9. Here, we confirmed ploidy increase in HEL, corroborated by increased expression of cyclin D, a cell cycle regulator whose overexpression increases MEG ploidy 56. Early stages of megakaryopoiesis are associated with an increase in cell cycle regulators, and initial low expression of genes involved in MEG terminal differentiation 57, 58. This pattern of expression correlates with the pattern seen in HEL, where ID induces ploidy, while genes involved in terminal differentiation are diminished. There was also upregulation of *ELF‐1*, a transcription factor progressively upregulated in megakaryopoiesis and downregulated in erythroid terminal differentiation 48, 49, and downregulation in beta hemoglobin, supporting the shift away from erythropoiesis. Further transcription factors involved in differentiation were also regulated, however only in HEL.

Rats on ID‐diet develop thrombocytosis, with bone marrow alterations suggesting not only increased MEG ploidy, but also MEG progenitor expansion, and enhanced differentiation 9. This matches our observed expansion of the MEG population expressing early and late markers of megakaryopoiesis, and enhanced proplatelet formation in both CBHSC and CMK. Furthermore, the proportion of MEGs readily producing proplatelets is increased in ID. Ploidy was not increased in CBHSCs, but it is known that cord blood–derived MEGs do not become highly polyploid 30 and ID‐enhanced endomitosis may still occur in bone marrow. CMK is a more mature cell line than HEL 41, 42 and, hence, exhibited induction of later features of megakaryopoiesis instead of ploidy. These changes are accompanied by the expression of *CD61* and *vWF*, which increase in terminal differentiation 58. While we did not see regulation of these genes in MEGs in these genes, this is likely a limitation of prior sorting for CD61 expression.

Previous studies suggest that TPO, IL‐6, and IL‐11 may not be the primary mediators of ID‐augmented megakaryopoiesis 9, 24. We identified VEGF as a potential pathway involved, as *VEGFR2* and *VEGFA* were modulated by ID and immunoassay of cell media showed increased *VEGFA* concentrations in ID. Addition of VEGF to culture media also increases the percentage of cells expressing CD41, with no additive effect of ID. Stimulation of *VEGFR2* induces the expression of megakaryocytic markers 53, while *VEGFR1* increases MEG ploidy 51, 52. Megakaryoid and erythroid precursors both secrete VEGF, which may then stimulate MEG differentiation in an autocrine and paracrine fashion 51, 53. Thus, ID could increase both receptor and ligand expression, leading to augmented MEG polyploidization and differentiation. Furthermore, stimulation of this pathway has been shown to increase the number of circulating platelets *in vivo*, by way of increased MEG migration toward sinusoids in the bone marrow 52.

HIFs are central regulators of the response to hypoxic stress. These transcription factors are regulated by the availability of iron and oxygen, two factors diminished in ID anemia. Furthermore, HIFs are known regulators of the VEGF pathway 54, 55, and HIF2α is also known to regulate cyclin D 59. Another important HIF2α target is EPO, allowing regulation of erythropoiesis and iron absorption in response to changes in oxygen and iron 60. We hypothesize that alterations in this pathway promote megakaryopoiesis. As MEGs retain HIF2α expression in ID, this could allow sustained expression of VEGFA, which facilitates megakaryopoiesis and MEG migration toward sinusoids, while erythropoiesis is stalled due to lack of iron for hemoglobin.

This study warrants further investigations into the HIF/VEGF pathways under ID. Our *in vitro* model does not take into account the bone microenvironment, which is involved in the regulation of megakaryopoiesis. Furthermore, ID *in vivo* progressively influences oxygenation and, while it is striking that ID alone can invoke such changes, the situation *in vivo* is likely more complex. In fact, hypoxia alone modulates megakaryopoiesis in rats, leading to increases in MEG volume, number, platelet count, and platelet aggregability 61. Thus, ID and hypoxia may work in concert to augment megakaryopoiesis. ID may also influence intracellular redox balance, and megakaryopoiesis as well as hematopoiesis in general are greatly influenced by reactive oxygen species production (see 62, 63 for reviews).

In conclusion, ID has a direct effect on megakaryopoiesis, augmenting MEG ploidy, as well as overall differentiation and subsequent proplatelet production. ID modulates gene expression, leading to increased HIF2α and increased expression of *VEGFA*. This would, in theory, lead to increased platelet production that would facilitate coagulation in the context of chronic bleeding. However, this could also increase the risk of thromboembolic events in patients suffering from chronic ID, particularly in IBD or cancer. Further investigation into megakaryopoiesis in ID is necessary.

## Addendum

K. Jimenez: experimental design, performed experiments, author of manuscript. V. Khare: experimental design, critical review of manuscript. R. Evstatiev: experimental design, performed animal experiments, statistical analysis, critical review of manuscript. S. Kulnigg‐Dabsch: cell culture, critical review of manuscript. M. Jambrich: cell culture, critical review of manuscript. H. Strobl: provided CBHSCs, critical review of manuscript. C. Gasche: conceptualization of study design, recipient of funding, editor of manuscript.

## Disclosure of Conflict of Interest

C. Gasche reports grants from AOP Pharmaceuticals and Biogena, as well as personal fees and nonfinancial support from Vifor International, outside the submitted work. All other authors state that they have no conflict of interest.

## Supporting information


**Fig. S1.** Proplatelet quantification.
**Fig. S2.** Iron deficiency increases TfR1 expression in HEL and CMK.
**Fig. S3.** Iron deficiency does not augment the effect of PMA.
**Fig. S4.** Iron deficiency increases the population of cells expressing megakaryocyte markers without altering ploidy.
**Fig. S5.** Iron deficiency increases percentage of CD41‐expressing cells.
**Fig. S6.** (A) White blood cell count (WBC), red blood cell count (RBC), and hematocrit (HCT) of control (*n* = 7) and iron‐deficient (*n* = 8) rats after 3 weeks.

**Table S1.** ΔΔ*C*_t_ of all investigated targets on qPCR arrays

**Data S1.** Supplementary methods.
