# Supplementary material for: Increased expression of HIF2α during iron deficiency–associated megakaryocytic differentiation
Source: J Thromb Haemost. 2015 Apr 23;13(6):1113–27. doi: 10.1111/jth.12884 (PMC4949661; doi:10.1111/jth.12884)
Supplement: Supplementary file 1 — Fig. S1. Proplatelet quantification. Fig. S2. Iron deficiency increases TfR1 expression in HEL and CMK. Fig. S3. Iron deficiency does not augment the effect of PMA. Fig. S4. Iron deficiency increases the population of cells expressing megakaryocyte markers without altering ploidy. Fig. S5. Iron deficiency increases percentage of CD41‐expressing cells. Fig. S6. (A) White blood cell count (WBC), red blood cell count (RBC), and hematocrit (HCT) of control (n = 7) and iron‐deficient (n = 8) rats after 3 weeks. [file JTH-13-1113-s001.pdf]

A.

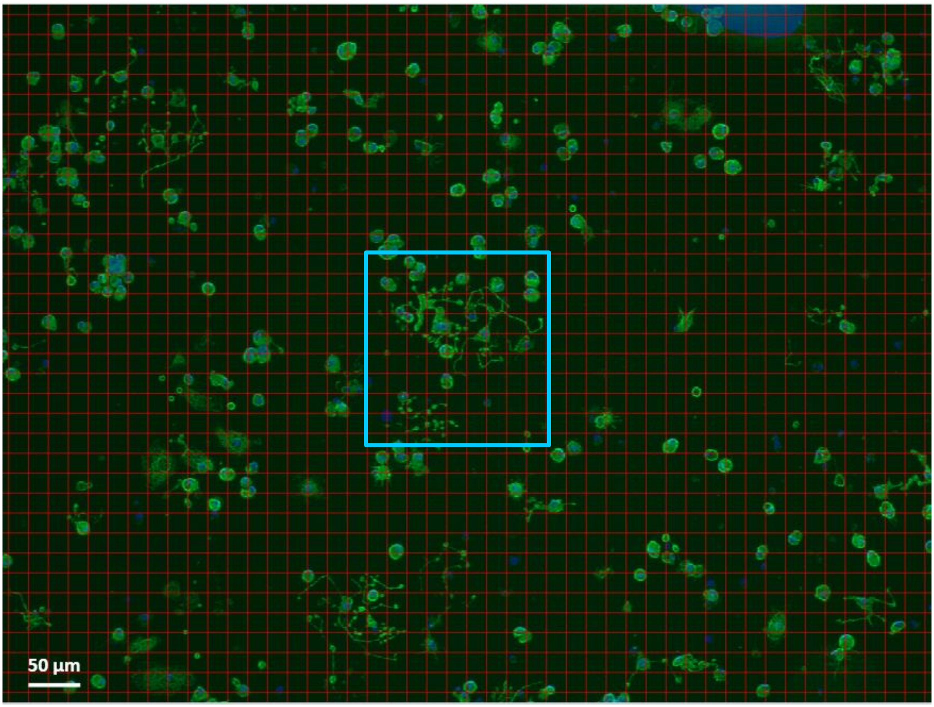

B.

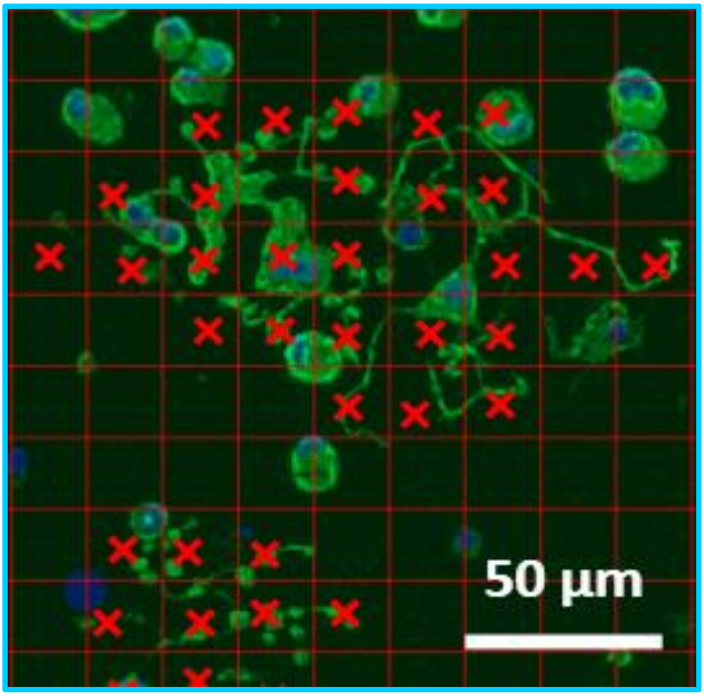

Figure S1. Proplatelet quantification.

Proplatelet quantification was performed by superimposing a 20x20µm grid on acquired images (A) and each square on the grid enclosing a proplatelet was counted as one unit (B, inset zoomed). Analysis was performed on Zen lite software (Zeiss). Total cell counts as well as number of megakaryocytes forming proplatelets were also counted per image.

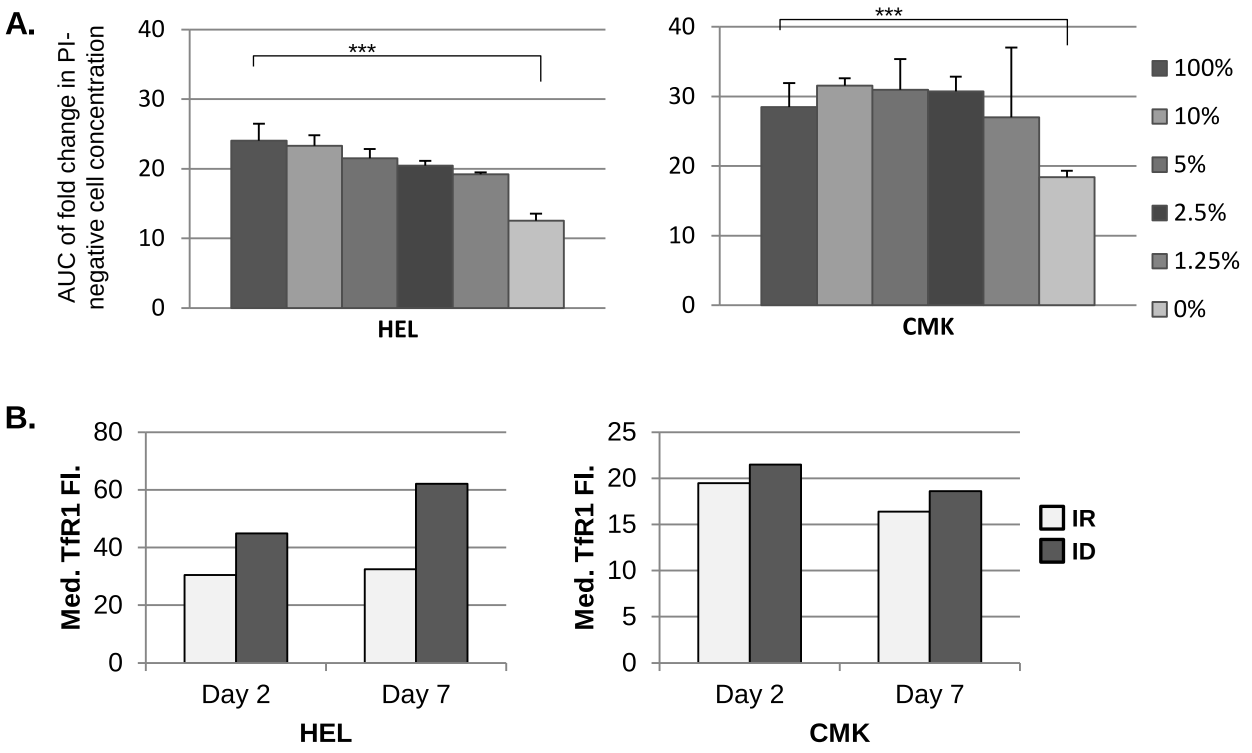

Figure S2. ID increases TfR1 expression in HEL and CMK

(A) The area under the curve (AUC) was calculated for each condition and experiment using the formula AUC= Σ [(ValueT1 + ValueT2) * time(T2-T1)/2]. Graph depicts the mean calculated AUC from Figure 1, of HEL and CMK cells upon culture in 100%, 10%, 5%, 2.5%, 1.25%, and 0% v/v Panserin 401 in Panserin 401S after from 0-7 days of culture. 0% was significantly different from all other conditions by ANOVA with Tukey post-hoc testing. *** $p \leq 0.001$

(B) Median TfR1 fluorescence (CD71, Exbio, Prague) on flow cytometric measurement of HEL and CMK after culture in iron deficiency for 2 and 7 days in iron replete and iron deficient medium. Results from 1-3 independent experiments are shown.

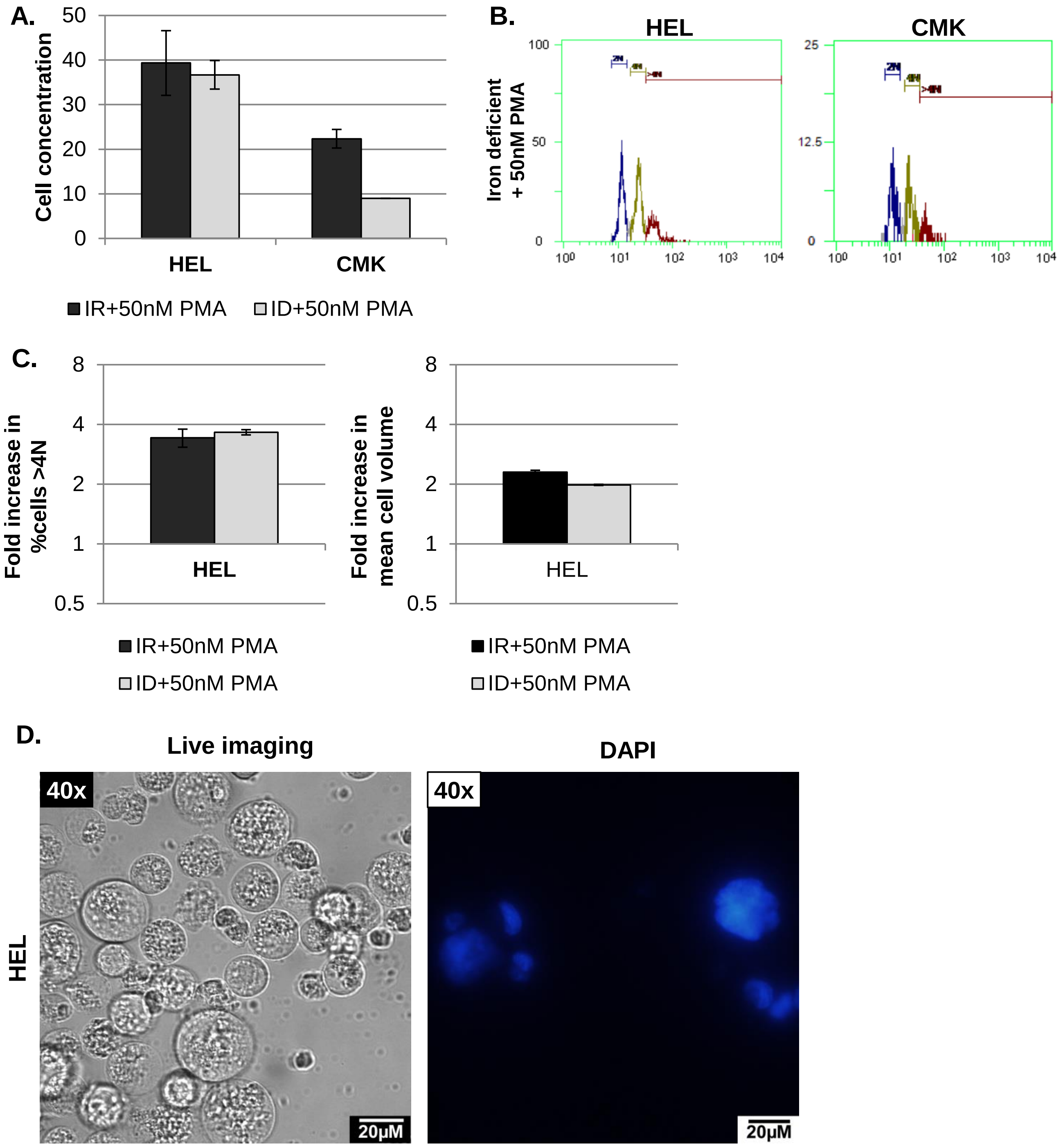

Figure S3. ID does not augment the effect of PMA. (A) HEL and CMK cultured for 4 days in IR and ID plus 50nM PMA. Graph depicts the cell concentrations measured by flow cytometry, with a decrease in CMK but not in HEL. (B) Representative histograms of ploidy in HEL and CMK after 4 days culture in ID+50nM PMA. (C) Flow cytometric measurement of Hoechst 33342 nuclear staining and cell size of HEL. Graphs depict the percentage of cells with ploidy greater than 4n as a fold change to IR. (D) Representative live images and nuclear staining of HEL after 4 days treatment with ID plus 50nM of PMA. The results from 2-3 independent experiments are shown.

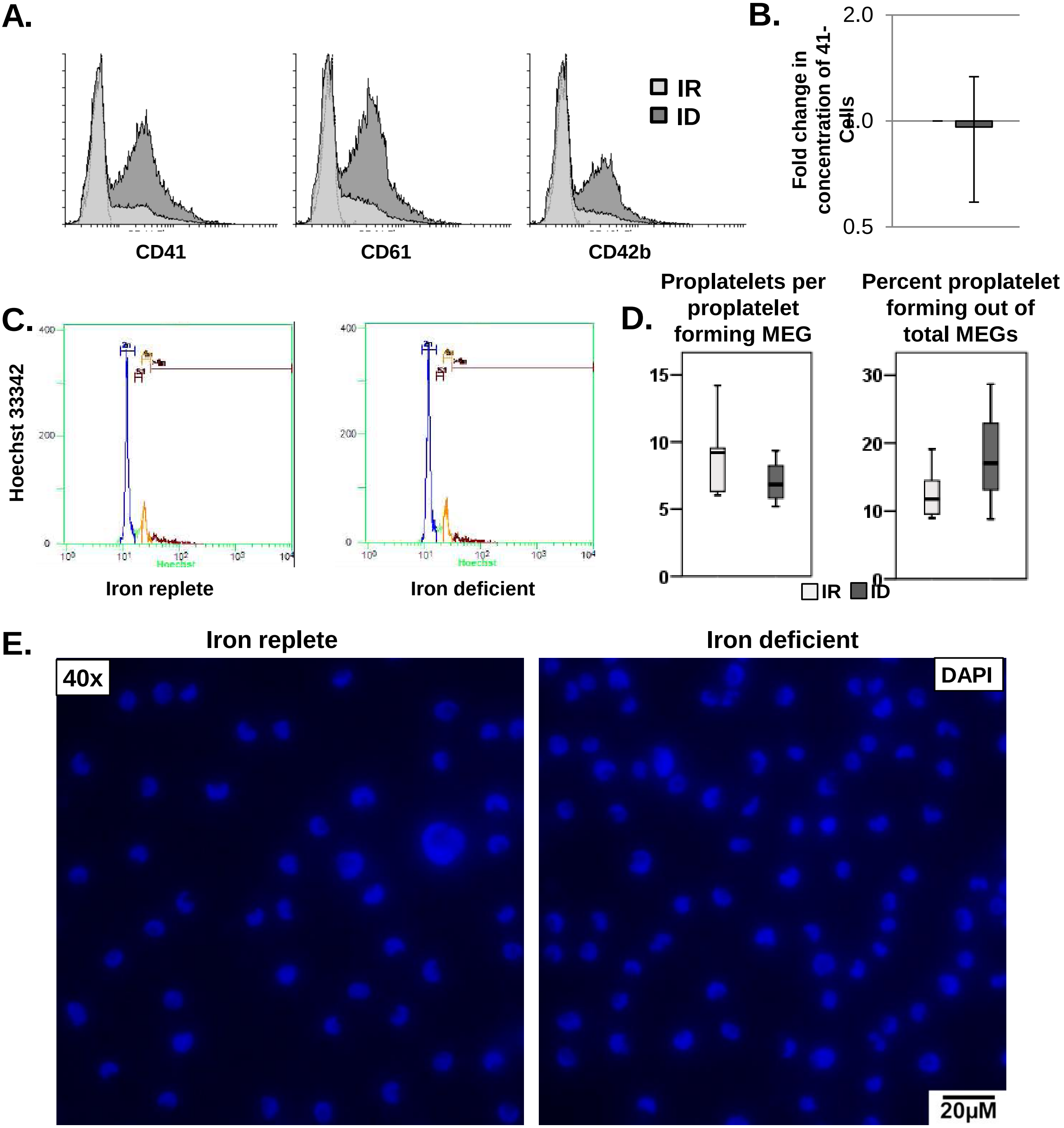

Figure S4. Iron deficiency increases the population of cells expressing megakaryocyte markers without altering ploidy. (A) Representative histograms of CD41, CD61, and CD42b median fluorescence intensity in cord blood derived hematopoietic stem cells after 5 days of treatment in IR and ID medium (B) Fold change in the concentration of CD41- negative cells in ID as compared to IR medium after 5 days of treatment. (C) Representative histograms of ploidy. (D) Ratio of proplatelet number to number of proplatelet forming MEGs and percent of proplatelet forming MEGs out of total MEGs. (E) Representative images of nuclear staining in ID and IR medium. Results are representative of 3-5 experiments.

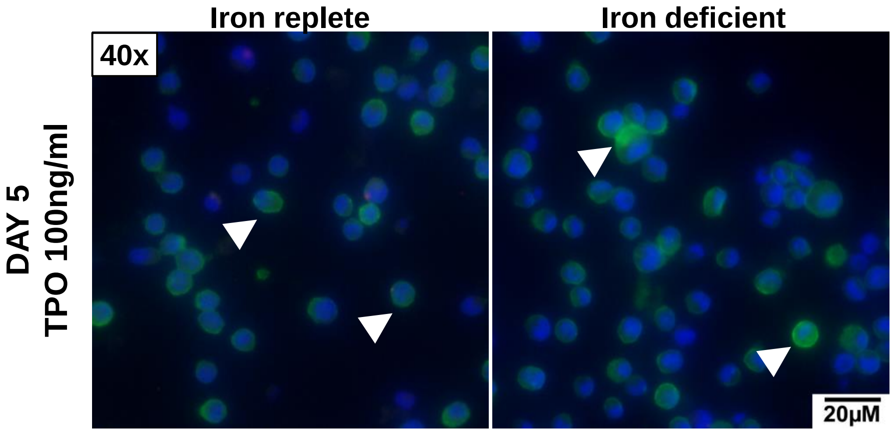

Figure S5. Iron deficiency increases percentage of CD41-expressing cells. Representative images of CD41 (green, arrowheads) and DAPI nuclear staining after culture in IR and ID media supplemented with 100ng/ml TPO for five days.

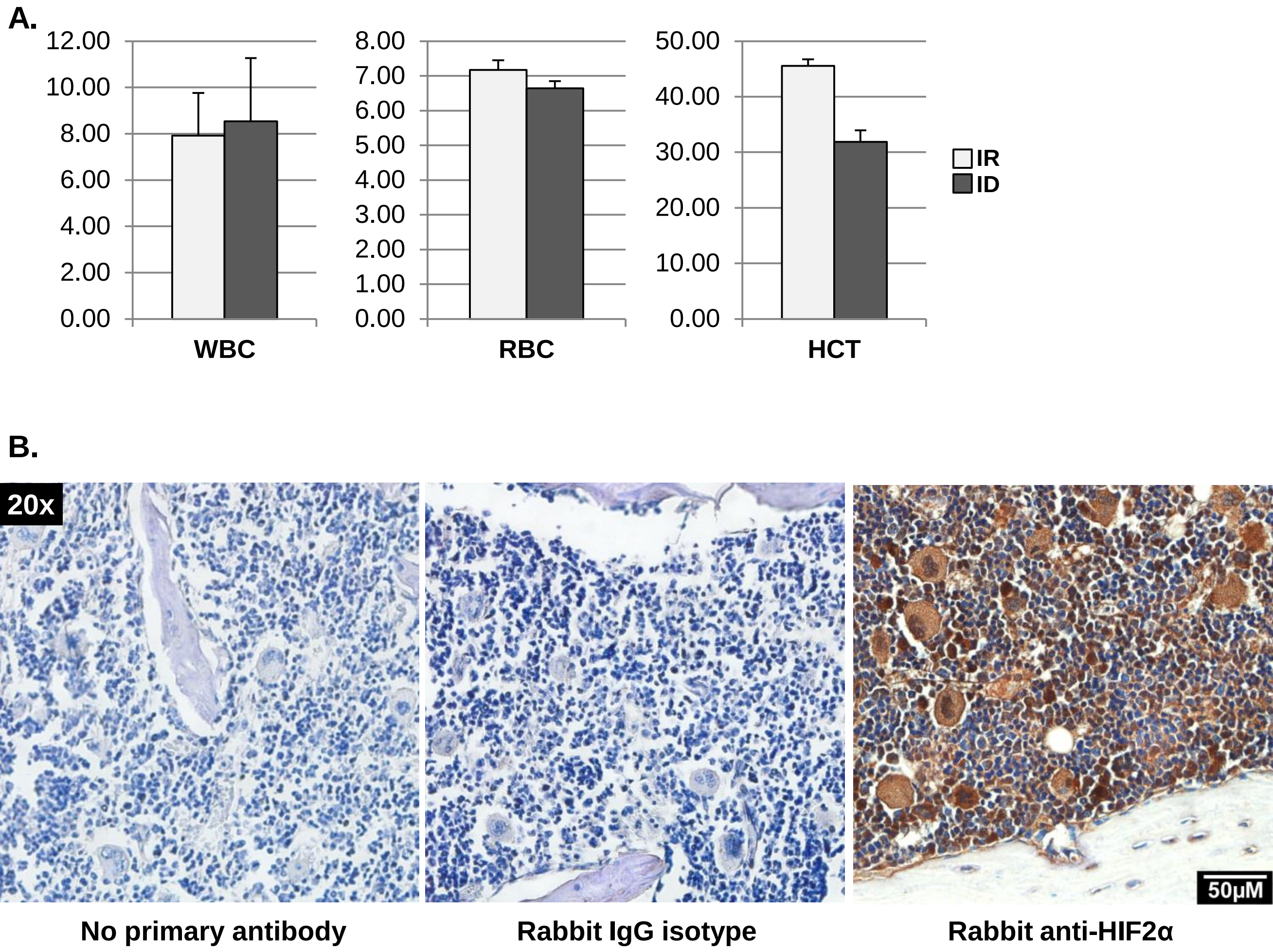

Figure S6. (A) White blood cell count (WBC), Red blood cell count (RBC), and hematocrit (HCT) of control (n=7) and iron deficient (n=8) rats after 3 weeks. Analysis was performed on a a Cell-Dyn 3500 analyzer. (B) Rat bone marrow sections stained using DAB-HRP without primary antibody, with a rabbit IgG isotype control, or with rabbit anti-HIF2α (Novus Biologicals).
