# Supplementary material for: Increased expression of HIF2α during iron deficiency–associated megakaryocytic differentiation
Source: J Thromb Haemost. 2015 Apr 23;13(6):1113–27. doi: 10.1111/jth.12884 (PMC4949661; doi:10.1111/jth.12884)
Supplement: Supplementary file 2 — Table S1. ΔΔC t of all investigated targets on qPCR arrays [file JTH-13-1113-s002.pdf]

SUPPLEMENTARY FIGURES

Supplementary table 1. ΔΔCt of all investigated targets on qPCR Arrays.

Upregulated	Downregulated	Not expressed

GROUP		GENE NAME	ΔΔCt			
			HEL	CMK	MEG	
Regulated Targets	Iron Homeostasis	TFR1	2.17	1.67	1.84	transferrin receptor (p90, CD71)
	Iron Homeostasis	FTH	2.89	0.98	0.90	ferritin, heavy polypeptide 1
	Iron Homeostasis	HO-1	0.61	0.89	2.10	heme oxygenase (decycling) 1
	HIF/VEGF	HIF2α	2.25	1.13	2.03	endothelial PAS domain protein 1
	HIF/VEGF	VEGFR1	2.24	1.19	1.75	fms-related tyrosine kinase 1 (vascular endothelial growth factor/vascular permeability factor receptor)
	HIF/VEGF	VEGFR2	0.61	1.18	2.13	kinase insert domain receptor (a type III receptor tyrosine kinase)
	Hematopoiesis and Differentiation	HBB	0.48	1.06	0.94	hemoglobin, beta
	Hematopoiesis and Differentiation	VWF	0.24	1.80	0.98	von Willebrand factor
	Hematopoiesis and Differentiation	CD61	0.49	1.51	1.13	integrin, beta 3 (platelet glycoprotein IIIa, antigen CD61)
	Hematopoiesis and Differentiation	PPBP	0.14	1.30	1.12	pro-platelet basic protein (chemokine (C-X-C motif) ligand 7)
	Hematopoiesis and Differentiation	PF4	0.16	1.02	1.08	platelet factor 4
	Hematopoiesis and Differentiation	CXCR4	0.30	1.46	1.25	chemokine (C-X-C motif) receptor 4
	Hematopoiesis and Differentiation	ELF-1	6.70	1.19		ephrin-A2
	Cell Cycle/Apoptosis	CCND1	7.17	0.79	0.95	cyclin D1
	Cell Cycle/Apoptosis	BCL2	2.41	0.89	0.91	B-cell CLL/lymphoma 2
	Transcription Factors	GATA6	10.14	1.03		GATA binding protein 6
	Transcription Factors	AR	5.14	0.98	0.90	androgen receptor
	Transcription Factors	ATF3	2.49	0.72	0.63	activating transcription factor 3
	Transcription Factors	FOS	0.26	0.84	0.75	v-fos FBJ murine osteosarcoma viral oncogene homolog
	Transcription Factors	FOXO1	5.66	1.46	1.01	forkhead box O1
	Transcription Factors	FOXA2	6.10		0.96	forkhead box A2
	Transcription Factors	SMAD9	6.78		0.79	SMAD family member 9
	Transcription Factors	SMAD1	9.17	1.07	1.06	SMAD family member 1
	Transcription	TGIF1	4.82	1.11	0.86	TGFB-induced factor homeobox 1

	Factors					
Non-regulated Targets	Iron Homeostasis	FTMT			1.07	ferritin mitochondrial
	Iron Homeostasis	SLC11A1			1.07	solute carrier family 11 (proton-coupled divalent metal ion transporters), member 1
	Iron Homeostasis	HAMP	1.09	1.13		hepcidin antimicrobial peptide
	Iron Homeostasis	TWSG1	1.14	0.96	1.09	twisted gastrulation homolog 1 (Drosophila)
	Iron Homeostasis	FECH	1.19	1.14	1.12	ferrochelatase
	Iron Homeostasis	ALAS1	1.21	1.15	1.20	aminolevulinate, delta-, synthase 1
	Iron Homeostasis	TFR2	1.22	1.26	1.24	transferrin receptor 2
	Iron Homeostasis	ALAS2	1.23	1.43	1.07	aminolevulinate, delta-, synthase 2
	Iron Homeostasis	FXN	1.43	0.91	0.89	frataxin
	Iron Homeostasis	ABCB7	1.67	1.05	1.15	ATP-binding cassette, sub-family B (MDR/TAP), member 7
	Iron Homeostasis	HMOX2	1.69	0.90	1.11	heme oxygenase (decycling) 2
	Iron Homeostasis	FLVCR1	1.74	0.91	1.16	feline leukemia virus subgroup C cellular receptor 1
	Iron Homeostasis	HSD3B1				hydroxy-delta-5-steroid dehydrogenase, 3 beta- and steroid delta-isomerase 1
	Iron Homeostasis	SCARA 5				scavenger receptor class A, member 5 (putative)
	Iron Homeostasis	ACO1	0.90	0.89	1.13	aconitase 1, soluble
	Iron Homeostasis	BACH1	0.81	1.19	1.27	BTB and CNC homology 1, basic leucine zipper transcription factor 1
	Iron Homeostasis	BMP6	0.80	0.94	1.38	bone morphogenetic protein 6
	Iron Homeostasis	EIF2AK1	1.50	1.14	1.09	eukaryotic translation initiation factor 2-alpha kinase 1
	Iron Homeostasis	FTL	1.03	1.15	1.09	ferritin, light polypeptide
	Iron Homeostasis	GDF15	1.19	1.06	1.16	growth differentiation factor 15
	Iron Homeostasis	IREB2	1.06	0.95	1.20	iron-responsive element binding protein 2
	Iron Homeostasis	NRF1	1.00	1.07	1.23	nuclear respiratory factor 1
	Iron Homeostasis	SLC22A17	0.81	1.33	0.94	solute carrier family 22, member 17
	Iron Homeostasis	SLC25A37	1.06	1.27	1.09	solute carrier family 25, member 37
	Iron Homeostasis	SLC40A1	1.18	1.69	1.34	solute carrier family 40 (iron-regulated transporter), member 1
	HIF/VEGF	VEGFA	1.41	1.17	1.25	vascular endothelial growth factor A
	HIF/VEGF	VEGFB	1.63	1.10	1.10	vascular endothelial growth factor B
	Oxidative stress	NOS2	0.67	0.94	1.07	nitric oxide synthase 2, inducible
	Oxidative stress	CAT	1.33	1.04	1.12	catalase
	Oxidative stress	GPX4	1.26	1.13	1.24	glutathione peroxidase 4 (phospholipid hydroperoxidase)
	Oxidative stress	SOD1	1.00	0.90	0.96	superoxide dismutase 1, soluble
	Oxidative stress	SOD2	1.04	1.15	1.15	superoxide dismutase 2, mitochondrial
	Hematopoiesis and Differentiation	GP6	0.53	1.03	1.07	glycoprotein VI (platelet)
	Hematopoiesis and Differentiation	GPX1	0.55	1.23	0.89	glutathione peroxidase 1
	Hematopoiesis and Differentiation	CD34	0.57	1.13	1.45	CD34 molecule
	Hematopoiesis	TUBB1	0.60	1.68	1.21	tubulin, beta 1

	**and Differentiation**					
	**Hematopoiesis and Differentiation**	**CD41**	0.63	1.10	1.02	integrin, alpha 2b (platelet glycoprotein IIb of IIb/IIIa complex, antigen CD41)
	**Hematopoiesis and Differentiation**	**GP5**	0.65	1.12	1.14	glycoprotein V (platelet)
	**Hematopoiesis and Differentiation**	**CD42b**	0.72	1.03	0.96	glycoprotein Ib (platelet), alpha polypeptide
	**Hematopoiesis and Differentiation**	**S1PR4**	0.73	1.07	0.92	sphingosine-1-phosphate receptor 4
	**Hematopoiesis and Differentiation**	**CD36**	0.74	1.32	1.32	CD36 molecule (thrombospondin receptor)
	**Hematopoiesis and Differentiation**	**MPL**	0.75	1.36	1.23	myeloproliferative leukemia virus oncogene
	**Hematopoiesis and Differentiation**	**EPOR**	0.84	1.41	0.97	erythropoietin receptor
	**Hematopoiesis and Differentiation**	**CXCL12**	0.97		1.17	chemokine (C-X-C motif) ligand 12
	**Hematopoiesis and Differentiation**	**EPO**	1.09	1.13		erythropoietin
	**Hematopoiesis and Differentiation**	**GYPA**	1.42	1.40	0.97	glycophorin A (MNS blood group)
	**Hematopoiesis and Differentiation**	**THPO**	1.96	1.01		thrombopoietin
	**Hematopoiesis and Differentiation**	**JAK2**	0.82	1.21	1.29	Janus kinase 2
	**Hematopoiesis and Differentiation**	**KIT**	0.88	0.94	0.99	v-kit Hardy-Zuckerman 4 feline sarcoma viral oncogene homolog
	**Hematopoiesis and Differentiation**	**KITLG**	1.65	1.02	0.74	KIT ligand
	**Hematopoiesis and Differentiation**	**SPI1**	0.92	1.20	1.08	spleen focus forming virus (SFFV) proviral integration oncogene spi1
	**Hematopoiesis and Differentiation**	**TYK2**	1.17	1.11	1.01	tyrosine kinase 2
	**Cell Cycle/Apoptosis**	**CDKN2A**			1.28	cyclin-dependent kinase inhibitor 2A (melanoma, p16, inhibits CDK4)
	**Cell Cycle/Apoptosis**	**BCL2L1**	0.75	1.19	1.21	BCL2-like 1
	**Cell Cycle/Apoptosis**	**BAX**	1.01	1.05	1.17	BCL2-associated X protein
	**Cell Cycle/Apoptosis**	**CCND3**	1.02	1.10	0.98	cyclin D3
	**Cell Cycle/Apoptosis**	**CDC6**	1.21	0.90	1.11	cell division cycle 6 homolog (S. cerevisiae)
	**Cell Cycle/Apoptosis**	**CDKN1A**	1.72	1.49	1.34	cyclin-dependent kinase inhibitor 1A (p21, Cip1)
	**Cell Cycle/Apoptosis**	**CCNE1**	1.78	0.99	1.24	cyclin E1
	**Cell Cycle/Apoptosis**	**CCNB1**	1.40	0.92	1.20	cyclin B1
	**Cell Cycle/Apoptosis**	**CDK1**	1.38	1.01	0.97	cyclin-dependent kinase 1
	**Cell Cycle/Apoptosis**	**CDK2**	1.19	1.09	1.14	cyclin-dependent kinase 2
	**Cell**	**CDKN1B**	1.08	1.21	1.07	cyclin-dependent kinase inhibitor 1B

	**Cycle/Apoptosis**					(p27, Kip1)
	**Cell Cycle/Apoptosis**	**MCM2**	0.86	0.91	1.09	minichromosome maintenance complex component 2
	**Transcription Factors**	**MEIS1**	0.63	1.19	1.20	Meis homeobox 1
	**Transcription Factors**	**HOXA9**	0.70	0.83	0.78	homeobox A9;HOXA10-HOXA9 readthrough
	**Transcription Factors**	**FOXO3**	0.89	1.19	1.26	forkhead box O3;forkhead box O3B pseudogene
	**Transcription Factors**	**HOXA11**	0.90	0.82	1.07	homeobox A11
	**Transcription Factors**	**CBFB**	1.43	0.99	1.19	core-binding factor, beta subunit
	**Transcription Factors**	**ELK4**	0.97	0.95	0.92	ELK4, ETS-domain protein (SRF accessory protein 1)
	**Transcription Factors**	**FLI1**	0.70	1.12	1.05	Friend leukemia virus integration 1
	**Transcription Factors**	**GABPA**	1.17	1.03	1.15	GA binding protein transcription factor, alpha subunit 60kDa
	**Transcription Factors**	**HOXB4**	1.45	1.06	0.89	homeobox B4
	**Transcription Factors**	**NFE2**	0.92	1.21	1.20	nuclear factor (erythroid-derived 2), 45kDa
	**Transcription Factors**	**NFE2L3**	0.73	0.89	1.20	nuclear factor (erythroid-derived 2)-like 3
	**Transcription Factors**	**RAB27B**	0.71	0.85	1.10	RAB27B, member RAS oncogene family
	**Transcription Factors**	**RUNX1**	0.76	1.12	1.12	runt-related transcription factor 1
	**Transcription Factors**	**TAL1**	0.71	1.04	1.21	T-cell acute lymphocytic leukemia 1
	**Transcription Factors**	**ZFPM1**	0.78	1.29	1.10	zinc finger protein, multitype 1
	**Transcription Factors**	**ARNT**	0.88	1.12	1.02	aryl hydrocarbon receptor nuclear translocator
	**Transcription Factors**	**ATF1**	0.88	0.88	1.14	activating transcription factor 1
	**Transcription Factors**	**ATF2**	0.75	1.03	1.14	activating transcription factor 2
	**Transcription Factors**	**ATF4**	0.72	0.97	1.24	activating transcription factor 4 (tax-responsive enhancer element B67)
	**Transcription Factors**	**B2M**	0.59	1.31	0.94	beta-2-microglobulin
	**Transcription Factors**	**CEBPA**	0.98	0.89	1.19	CCAAT/enhancer binding protein (C/EBP), alpha
	**Transcription Factors**	**CEBPB**	1.23	0.98	1.13	CCAAT/enhancer binding protein (C/EBP), beta
	**Transcription Factors**	**CEBPG**	1.14	0.93	1.28	CCAAT/enhancer binding protein (C/EBP), gamma
	**Transcription Factors**	**CREB1**	0.74	1.30	1.00	cAMP responsive element binding protein 1
	**Transcription Factors**	**CREBBP**	0.83	1.29	0.96	CREB binding protein
	**Transcription Factors**	**CTNNB1**	0.76	1.09	1.32	catenin (cadherin-associated protein), beta 1, 88kDa

	**Transcription Factors**	**DR1**	0.78	0.96	1.03	down-regulator of transcription 1, TBP-binding (negative cofactor 2)
	**Transcription Factors**	**E2F1**	0.77	0.99	0.82	E2F transcription factor 1
	**Transcription Factors**	**E2F6**	0.86	0.98	1.01	E2F transcription factor 6
	**Transcription Factors**	**EGR1**	0.60	1.03	0.68	early growth response 1
	**Transcription Factors**	**ELK1**	1.13	1.15	1.05	ELK1, member of ETS oncogene family
	**Transcription Factors**	**ESR1**				estrogen receptor 1
	**Transcription Factors**	**ETS1**	0.55	1.14	1.41	v-ets erythroblastosis virus E26 oncogene homolog 1 (avian)
	**Transcription Factors**	**ETS2**	0.57	1.25	1.06	v-ets erythroblastosis virus E26 oncogene homolog 2 (avian)
	**Transcription Factors**	**GATA1**	0.69	1.17	0.99	GATA binding protein 1 (globin transcription factor 1)
	**Transcription Factors**	**GATA2**	0.65	1.06	1.13	GATA binding protein 2
	**Transcription Factors**	**GATA3**	0.70	0.87	1.12	GATA binding protein 3
	**Transcription Factors**	**GTF2B**	0.77	1.20	1.02	general transcription factor IIB
	**Transcription Factors**	**GTF2F1**	0.84	0.94	0.94	general transcription factor IIF, polypeptide 1, 74kDa
	**Transcription Factors**	**GUSB**	1.30	1.06	1.19	glucuronidase, beta
	**Transcription Factors**	**HAND1**			0.96	heart and neural crest derivatives expressed 1
	**Transcription Factors**	**HAND2**				heart and neural crest derivatives expressed 2
	**Transcription Factors**	**HDAC1**	0.82	0.98	1.54	histone deacetylase 1
	**Transcription Factors**	**HIF1A**	0.71	1.15	1.57	hypoxia inducible factor 1, alpha subunit (basic helix-loop-helix transcription factor)
	**Transcription Factors**	**HMBS**	0.77	1.00	0.98	hydroxymethylbilane synthase
	**Transcription Factors**	**HNF1A**				HNF1 homeobox A
	**Transcription Factors**	**HNF4A**	0.82	1.36	0.59	hepatocyte nuclear factor 4, alpha
	**Transcription Factors**	**HOXA5**	0.63	1.05	1.97	homeobox A5
	**Transcription Factors**	**HSF1**	0.77	0.90	0.94	heat shock transcription factor 1
	**Transcription Factors**	**ID1**	0.58	1.02	1.40	inhibitor of DNA binding 1, dominant negative helix-loop-helix protein
	**Transcription Factors**	**IRF1**	0.58	1.28	0.89	interferon regulatory factor 1
	**Transcription Factors**	**JUN**	1.40	0.70	0.75	jun oncogene
	**Transcription Factors**	**JUNB**	0.61	0.82	0.82	jun B proto-oncogene

	**Transcription Factors**	**JUND**				jun D proto-oncogene
	**Transcription Factors**	**MAX**	0.79	1.33	0.97	MYC associated factor X
	**Transcription Factors**	**MEF2A**	0.67	1.30	0.93	myocyte enhancer factor 2A
	**Transcription Factors**	**MEF2B**	1.07	1.01	1.17	myocyte enhancer factor 2B
	**Transcription Factors**	**MEF2C**	0.58	1.25	1.47	myocyte enhancer factor 2C
	**Transcription Factors**	**MYB**	0.90	1.13	1.11	v-myb myeloblastosis viral oncogene homolog (avian)
	**Transcription Factors**	**MYC**	0.79	1.20	1.05	v-myc myelocytomatosis viral oncogene homolog (avian)
	**Transcription Factors**	**MYF5**				myogenic factor 5
	**Transcription Factors**	**MYOD1**				myogenic differentiation 1
	**Transcription Factors**	**NFAT5**	1.10	1.19	0.99	nuclear factor of activated T-cells 5, tonicity-responsive
	**Transcription Factors**	**NFATC1**	1.35	1.09	1.01	nuclear factor of activated T-cells, cytoplasmic, calcineurin-dependent 1
	**Transcription Factors**	**NFATC2**	0.74	1.06	1.02	nuclear factor of activated T-cells, cytoplasmic, calcineurin-dependent 2
	**Transcription Factors**	**NFATC3**	0.86	1.05	1.02	nuclear factor of activated T-cells, cytoplasmic, calcineurin-dependent 3
	**Transcription Factors**	**NFATC4**	0.64	1.16	0.91	nuclear factor of activated T-cells, cytoplasmic, calcineurin-dependent 4
	**Transcription Factors**	**NFKB1**	0.84	0.97	0.95	nuclear factor of kappa light polypeptide gene enhancer in B-cells 1
	**Transcription Factors**	**NFYB**	0.84	0.97	1.04	nuclear transcription factor Y, beta
	**Transcription Factors**	**NR3C1**	1.34	1.26	1.06	nuclear receptor subfamily 3, group C, member 1 (glucocorticoid receptor)
	**Transcription Factors**	**PAX6**	0.97	1.09		paired box 6
	**Transcription Factors**	**PGK1**	0.99	1.00	1.05	phosphoglycerate kinase 1
	**Transcription Factors**	**POU2AF1**				POU class 2 associating factor 1
	**Transcription Factors**	**PPARA**	0.57	1.09	1.15	peroxisome proliferator-activated receptor alpha
	**Transcription Factors**	**PPARG**			0.89	peroxisome proliferator-activated receptor gamma
	**Transcription Factors**	**PPIA**	1.03	0.83	0.96	peptidylprolyl isomerase A (cyclophilin A)
	**Transcription Factors**	**RB1**	0.72	1.21	0.95	retinoblastoma 1
	**Transcription Factors**	**REL**	0.94	0.97	1.09	v-rel reticuloendotheliosis viral oncogene homolog (avian)
	**Transcription Factors**	**RELA**	0.86	0.99	0.93	v-rel reticuloendotheliosis viral oncogene homolog A (avian)
	**Transcription Factors**	**RELB**	0.91	1.16	0.77	v-rel reticuloendotheliosis viral oncogene homolog B

	**Transcription Factors**	**RPLP0**	0.84	1.02	1.08	ribosomal protein, large, P0
	**Transcription Factors**	**SMAD4**	0.88	1.16	1.16	SMAD family member 4
	**Transcription Factors**	**SMAD5**	0.93	1.01	0.96	SMAD family member 5
	**Transcription Factors**	**SP1**	0.86	1.17	1.04	Sp1 transcription factor
	**Transcription Factors**	**SP3**	0.90	1.09	1.28	Sp3 transcription factor
	**Transcription Factors**	**STAT1**	0.72	1.11	1.01	signal transducer and activator of transcription 1, 91kDa
	**Transcription Factors**	**STAT2**	0.72	1.00	0.94	signal transducer and activator of transcription 2, 113kDa
	**Transcription Factors**	**STAT3**	0.64	1.26	0.96	signal transducer and activator of transcription 3 (acute-phase response factor)
	**Transcription Factors**	**STAT4**	0.60	0.74	0.93	signal transducer and activator of transcription 4
	**Transcription Factors**	**STAT5A**	0.96	0.94	0.97	signal transducer and activator of transcription 5A
	**Transcription Factors**	**STAT5B**	1.02	0.93	0.94	signal transducer and activator of transcription 5B
	**Transcription Factors**	**STAT6**	0.91	1.15	1.09	signal transducer and activator of transcription 6, interleukin-4 induced
	**Transcription Factors**	**TBP (A9)**	1.41	1.16	1.01	TATA box binding protein
	**Transcription Factors**	**TBP (H6)**	0.92	1.13	1.17	TATA box binding protein
	**Transcription Factors**	**TCF7L2**	1.23	1.40	1.05	transcription factor 7-like 2 (T-cell specific, HMG-box)
	**Transcription Factors**	**TFAP2A**	1.65	1.26	1.00	transcription factor AP-2 alpha (activating enhancer binding protein 2 alpha)
	**Transcription Factors**	**TP53**	0.55	0.96	0.75	tumor protein p53
	**Transcription Factors**	**UBC**	0.97	1.18	1.01	ubiquitin C
	**Transcription Factors**	**YY1**	0.96	1.09	0.61	YY1 transcription factor
