# Supplementary material for: Increased expression of HIF2α during iron deficiency–associated megakaryocytic differentiation
Source: J Thromb Haemost. 2015 Apr 23;13(6):1113–27. doi: 10.1111/jth.12884 (PMC4949661; doi:10.1111/jth.12884)
Supplement: Supplementary file 3 — Data S1. Supplementary methods. [file JTH-13-1113-s003.pdf]

Supplementary Methods

Flow cytometric measurement of Megakaryocyte markers and ploidy

Expanded CBHSCs were cultured in IR or ID medium supplemented with 100ng-ml TPO for five days. Cells were then collected, blocked with 20% serum (Fisher Scientific) and stained with fluorescent-conjugated anti-CD41-PE-Cy7, anti-CD61-PE-Cy7, or anti-CD42b-FITC at 1:100 concentration. Percentage positive staining cells was determined using flow cytometry, and the median fluorescence intensity was based on the gated positive cell population of each megakaryocyte marker. To assess ploidy, cells were stained with both Hoechst 33342 (Invitrogen) and anti-CD41-PE-Cy7, and only the CD41-positive cells were included in histograms. Results were depicted as a fold change in ID values to IR values, and tested using a one-sample t-test with a test value of 1.

Cell counting upon VEGFA treatment

Expanded CBHSCs were cultured in IR or ID medium which were supplemented with either 25ng/ml VEGF (eBioscience), 1ng/mL erythropoietin (EPO, eBioscience) or 100ng/ml TPO. After 2 and 5 days, cells were collected and stained with 1:100 anti-CD41-FITC and 1:100 anti-glycophorin A-PE-Cy5 (erythrocyte) conjugated antibodies (eBioscience). Cells were adhered to poly-l-lysine coated slides and fixed with 4% paraformaldehyde (Sigma). Nuclei were counterstained with DAPI (Vectashield). Cells were counted under 40x magnification, adding up multiple FOVs until a total of at least 100 cells was reached. To calculate percentages, the number of green (CD41) or red (Glycophorin A) cells were divided by the total number of visible nuclei. For statistical testing, an ANOVA with Tukey post-hoc testing was performed.

Proplatelet formation

Equivalent amounts of cells were transferred at day 5 of culture to a glass coverslip pre-coated with 100µg/mLfibrinogen (Sigma) and incubated for 20 hours to allow adhesion of cells expressing fibrinogen receptor (CD41/61). After fixation and permeabilization, cells were blocked with 3% BSA in PBS, incubated with 1:100 mouse anti-α-tubulin (Sigma) followed by 1:1000 anti-mouse IgG conjugated to Alexa Fluor 488 (Invitrogen). DAPI was used for nuclear counterstaining (Vectashield). Images were taken using an Axioimager M2 (Zeiss). Each image corresponded to one field of view (FOV) at 10x magnification. To quantify proplatelet formation, a 20x20µm grid was superimposed on acquired images and each square on the grid enclosing a proplatelet was counted as one unit (Figure S1). Analysis was performed on Zen lite software (Zeiss). Total megakaryocyte counts as well as number of megakaryocytes forming proplatelets were counted per image.

Real time PCR analysis of gene expression

RNA was isolated using TRIzol® (Invitrogen) and cleaned with the RNeasy Mini Kit (Qiagen, Germany). RNA quantity was measured on a Nanodrop 1000 (PeqLab, Erlangen, Germany) and reverse transcribed using the High Capacity cDNA Reverse Transcription kit (Applied Biosystems, California, USA). All RT-PCR was run on the 7500 Fast Real-Time PCR System (Applied Biosystems). Analysis was performed using 7500 system software, DataAssist (Applied Biosystems), and LinRegPCR[38]. Relative expression was calculated using 2^-(CT_{Target}-,$CT_{Housekeeping}$) and relative quantity using 2^-(ΔCT_{ID}-ΔCT_{IR}).

A TaqMan RT-PCR array with customized targets, and a catalogued array for transcription factors (Applied Biosystems) were utilized for analysis of differential gene expression in ID(Supplementary Table 1). RNA quality (≥9.0) was evaluated using an Agilent 2100 Bioanalyzer (Agilent Technologies, California, USA). RNA was pooled from two independent experiments after one day (HEL) or two days (CMK) of culture in ID. For CD34+ cell cultures, CD61+ megakaryocytes (MEGs) were sorted using MACS (Miltenyi) after five days of culture, and RNA from three independent experiments was pooled. HPRT1 and 18S were used as endogenous controls. Cut-offs were placed at 2.0 for upregulation and 0.5 for downregulation.

The expression of individual genes such as TfR1 (TFRC, QT00094850, Qiagen), HIF2α (EPAS1, QT00069587), HIF1α (QT00083664), VEGFA (QT01682072), VEGFR1, and VEGFR2 (VBC Biotech) was performed with Fast SYBR Green Master Mix (Applied Biosystems). For TfR1, RNA was isolated after 1, 2, 4, and 7 days of ID in cell lines, and 5 days in MEGs. The expression of aforementioned genes were analysed after 3 days of ID in HEL and CMK, and 5 days ID in CD61-positive and CD61-negative cells.
